# Extraordinary

**Published:** 1870-04

**Authors:** 


### EXTRAORDINARY.

The most remarkable event of the times in the printing line, is, that a really good and permanently useful book should prove a "success." And a book to be a "success," according to

Gail Hamilton, who is no mean authority, is that five editions should be sold. We said, in the March number, of a new book published by Hurd & Houghton of New York and Boston, called

"HEALTH BY GOOD LIVING,"

that it was a very good book ; the public have endorsed our opinion by calling for the fifth edition within a month of the first issue. This proves most conclusively that the doleful vaticinations of all the old fogies of the time are but

"The baseless fabric of a vision,"

that instead of the world "progressing backward," everything good is "onward," as well as the star of empire. The people are really beginning to wake up to the proper appreciation of what is solid and useful and practical and true ; are beginning to find out that they have bodies ; that these bodies ought to be cared for as well as a fine coat or a new hat, and will last the longer for such care. This is a happy omen in the estimation of the physician, who knows full well that half the race die half a century sooner than they would do, if the science of health were studied as it ought to be.

One of the valuable points in this successful book is that the reason of some every-day precepts is made so plain and clear that a child can see and feel the truth of the same, and seeing and feeling it too, can never forget it to the latest hour of life. For example, turn to the article about eating fast, and its injurious effect on the system in causing life-long dyspeptic torments : a child in years, reading this, would be more vividly impressed with the reason why eating rapidly is injurious to health, than with ten thousand parental admonitions that they must not eat fast, because it injures the health, and is not " genteel."

Take another example, eating without an appetite; this is done every day; grown men and women, sensible men and women, sensible in other things, do it every day, and yet the commonest intellect cannot fail to feel the force of the illustration given on that subject, that it is such a violation of the laws of our being, that a persistence in it is wicked and is infinitely sure of harm. And ought we not to save the young, our own children, from life-long maladies by giving them some chance of learning how to take care of, and preserve the glorious health with which a beneficent Providence has endowed them? and if it can be done by the purchase of a dollar and a half book, it really seems that the omission to do so is a cruelty. On this very point, the *Christian Union*, edited by Henry Ward Beecher, and most likely his own words, says of " Health by Good Living,"—

A really good book. There is less flummery and more good sense in it than in any of the swarm of dietary books that have afflicted society. All young people from ten years of age to eighty would profit by reading it. We can cordially recommend it for school and village libraries, and if it could be made to kick out much of the trash which stuff an average Sunday-school library, it would do much toward teaching the young how to keep God's law written in their bodies.

From the *Sunday School Times* of Philadelphia.

Perhaps the stand-point of personal gratitude is not the most unprejudiced for the expression of an opinion, but it certainly ought to be the most hearty and sincere. We speak thus in commending Dr. Hall's book. We know what we say when we affirm the value of his views and general directions as to health and living. We have tested them happily in the past. Dr. Hall is no empiric. His good sense and wisdom have for years been spread before the public in his own *Journal of Health*, and in the thousand other journals that have quoted its articles; thousands have thus learned from him some of the simplest secrets of the art of living. Some of the good Doctor's directions seem to be dogmatic, and very dogmatically expressed, yet he only sets the standard high, and would bring men up to it. He does not believe in much "medicine." His "good living" means eating with a relish good food, well-cooked. Health can be maintained and disease cured by such living. The subjects treated in the book are, the object of eating, when, what, how, and how much to eat, regularity in eating, troubles from eating, air and exercise, food cure, etc. Families should by all means have such a good, plain, and sensible book of directions by them, know them by heart, and follow them.

The veteran *New York Observer* says:—

Dr. Hall is one of the most practical and sensible writers upon health of this age; and this book is like all his other works — clear, plain, and to the point. He shows the object of eating, when to eat, how to eat, and how much to eat; and gives rules which, if observed, would do much to prevent or cure biliousness, dyspepsia, neuralgia, and other diseases of which he has something to say. The argument of the book is, that high health can be maintained and ordinary diseases cured by eating with an appetite the best food prepared in the best manner; and he tells us what the best food is, how to cook it, and, above all, how to get and keep the good appetite, which is the *sine qua non* of high health.

From the *Congregationalist and Boston Recorder.*

Hall's *Journal of Health* has acquired fame for its energetic, pronounced, and common-sense suggestions in the matters of which it treats. Dr. Hall, its editor, has now issued a volume exhibiting the same characteristics, entitled "Health by Good Living," which we cordially commend to all who have any occasion to make their own health, or that of others, a subject of special consideration. We cannot help thinking, not only that a great many lives might be saved by the careful study of this manual, and good heed to its directions, but that many more might be lifted up from a plane of half-life to one of greatly increased vigor, comfort, and usefulness. We go for any doctor, who preaches plain food, fresh air, and much exercise out-of-doors.

From the *Michigan Farmer.*

This work is a practical and most excellent treatise on how to live and secure good health and an active body and mind, by giving some attention not only to what we eat, but how we eat. The author has set out with the design of informing his readers, in plain and clear terms, how they live, and by what means the system may be strengthened by the food which they consume. He first explains the object of eating, and then prescribes when eating should be practiced, and what should be eaten. He lays down no rules or regulations, but states in such a simple easily-understood language, and exemplifies his statements by such excellent practical remarks, that no one can doubt, not only the reasonableness of his recommendations, but must admit that he is correct, and has studied the laws of health and good living with great care and with ability to understand them. Probably no part of the work is of more value than the portion which treats of children, and how they should be regulated in their food, both as to quantity and to quality, and also as to the time when they should be permitted to eat. This treatise is also valuable to those who are troubled with biliousness, with dyspepsia, with neuralgia, and with debility which comes from a combination of all these disorders, and which arise principally from indigestion, and improper habits in the consumption of food, and from want of exercise. The book is one worthy of a place in every house, and should be read by every housekeeper; and Dr. Hall is entitled to a favorable hearing from the healthy as well as the sick.

From the *Chicago Advance.*

Dr. Hall is a preacher who does not propose to let the people go to sleep under his sermons. And so, while much that he says may not be especially new to us, it is so said that it is rarely uninteresting. He has shrewd common sense behind a good medical education and a large experience, and he contrives to pack into this volume not only a great deal of wisdom about eating and exercise, but a multitude of other things which interest humanity. He gives us opinions about social visits, novel reading, boarding-school morality, rocking chairs, Sunday employments, etc.., etc., though all somehow seem to be related to eating. He has some notions that will not find a wide acceptance, but for the most part his teachings are excellent. It is not often that a physician writes so large a book, in which there is not a word to send the reader to a medical dictionary; for which let the Doctor's newspaper experience be thanked.

The editor of the *Journal of Health* deserves the thanks of hundreds of his fellows whom he has materially aided in preserving and restoring one of the chief blessings of life, sound health. This volume is practical and useful, as might be anticipated from the character of its author. He would keep men healthy, not by drugs, but by attention to the laws of life, by intelligent and rational living. What man eats, how he eats, and when he eats, are of the highest importance to his well-being. Dr. Hall gives instruction on these points. He discusses the effects upon the system of different kinds of food; how they may be used to check tendencies to disease; their digestibility and nutritiousness. He says: —

"A man may have such a stock of high health as to resist the most destructive diseases. A drunkard is never well; a man who drinks largely is never well — is never in good health. It is a known fact that when the Asiatic cholera visits a community, its drunkards die first; the feeble of all classes die first. Sickness is debility, either of the whole body or of parts of it. Health is strength; they are absolutely inseparable. All human strength is derived from the food eaten, and can be derived from no other source. To keep up the strength, a man must eat, and he cannot do it in any other way. To keep up the strength is to keep the general health at a high point. It must then follow that to keep well we must eat well. To eat well we must have the best food, prepared in the best manner — that is, prepared in such a manner that the stomach may digest it most easily — and thus obtain the largest amount of nutriment with the least effort to the system. This is good living, in the sense in which the title of this book is used."

Other similar notices have been given by the press, and we have no hesitation whatever in taking up so much space in praise of our own book. We have got the praise and the "success," let that go; we now want something else more solid and substantial, something that is of some account, which is to suggest to parents that it is most likely a book spoken so well of by prominent educated men is a safe book for children to read. Suppose, for example, a father or mother has it in contemplation to prepare a son for the ministry, or, of greater moment still, for the missionary field in foreign lands, and that son, by reading the book, should fully understand the importance of taking care of his health, and should use the safe and easily practicable means for doing this, what arithmetic can compute the difference between the influences left on the world's history, from a broken constitution on the day of his leaving the seminary and travelling for his health, to die in a year or or two or less, and embarking for a foreign missionary field with a stalwart body and a vigorous intellect, and living to the age of a Judson, or a King, or a Morrison, working for the elevation of whole peoples from barbarism to the plane of a Bible Christianity. Parents! are these considerations nothing to you?

